# Periodontal Biology: Stem Cells, Bmp2 Gene, Transcriptional Enhancers, and Use of Sclerostin Antibody and Pth for Treatment of Periodontal Disease and Bone Loss

**DOI:** 10.16966/2472-6990.113

**Published:** 2017-01-27

**Authors:** Stephen E Harris, Michael Rediske, Rebecca Neitzke, Audrey Rakian

**Affiliations:** Department of Periodontics, University of Texas Health Science Center at San Antonio, San Antonio, TX 78229, USA

**Keywords:** Periodontium, Stem Cells, Epigenomics, Bmp2, Sclerostin, Pth

## Abstract

The periodontium is a complex tissue with epithelial components and a complex set of mesodermal derived alveolar bone, cellular and a cellular cementum, and tendon like ligaments (PDL). The current evidence demonstrates that the major pool of periodontal stem cells is derived from a population of micro vascular associated aSMA-positive stem/progenitor (PSC) cells that by lineage tracing form all three major mesodermal derived components of the periodontium. With *in vitro* aSMA+ stem cells, transcriptome and chip- seq experiments, the gene network and enhancer maps were determined at several differentiation states of the PSC. Current work on the role of the Bmp2 gene in the periodontal stem cell differentiation demonstrated that this Wnt regulated gene, Bmp2, is necessary for differentiation to all three major mesodermal derived component of the periodontium. The mechanism and use of Sclerostin antibody as an activator of Wnt signaling and Bmp2 gene as a potential route to treat craniofacial bone loss is discussed. As well, the mechanism and use of Pth in the treatment of periodontal bone loss or other craniofacial bone loss is presented in this review.

## Introduction

Periodontal disease is a major health problem that affects over 70% of the population in the US over 65 years of age. Efforts on regenerating the periodontal ligament have shown some limited success using periodontal ligament cells (PDL), with some accomplishments using cell homing techniques and in vivo growth factors such as GDF5 and BMPs [1–3]. If we can learn more about these periodontal stem cells (PSC), we may be able to use the patient’s own healing power to wake up, recruit, and activate the differentiation of these PSCs to regenerate tissues lost to periodontal disease, craniofacial defects, or trauma in general. This review will cover what we currently know about the complex differentiation process of mesoderm derived periodontal stem cells to tissues of the periodontium including the alveolar bone surrounding the tooth, the periodontal ligament that attaches bone to cementum, and the process of cementogenesis that is linked to formation of enthesis where the PDL integrates into the bone and cementum. We now know for example that many of the same genes and processes that control tendon and ligament formation in the rest of the body are also used by PSCs in their differentiation program. We also know that many of the genes expressed in cementum formation are expressed in osteoblast and osteocytes formation [4,5].

There is a linked between the healthy periodontium and root development and maintenance of the adjacent cementum and alveolar bone. For example, loss of the Periostin gene leads to highly disorganized PDL and major loss of associated alveolar bone [6–8]. Loss of the Bmp2 gene in Collagen 1 expressing cells in the early dental follicle and odontoblast precursors leads to profound loss of dentin and associated vascularization and associated mesenchymal stem cells (i.e. CD146+), as well as linked disruption of component of the periodontium, including alveolar bone [9]. However, as discussed below, new models that have inducible Cre activity in mesenchymal stem/progenitors cells were needed to separate at least partly, the issue of disrupted root formation indirectly altering the periodontal ligament and cementum formation, i.e. no root surface to attach PDL s).

**Figure 1:**
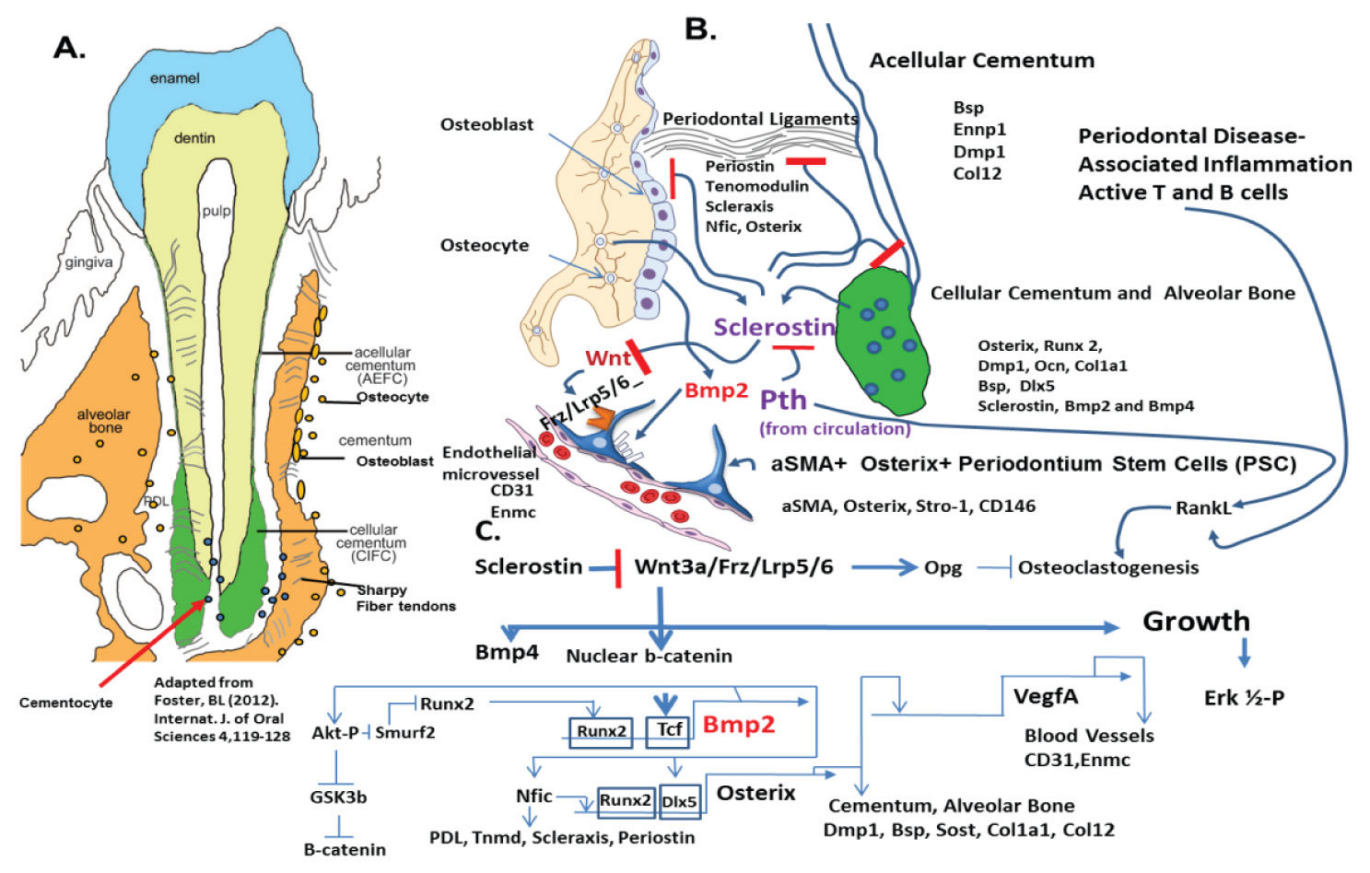
Model for Periodontium Biology and Molecular Pathways

Figure 1 presents a model of the many interacting genes and their proteins in periodontal biology and disease and potential targets for therapy.

Figure 1 Model of Interacting components and gene in formation and maintenance of the Periodontium. **(A)** Show overall components of the periodontium, with organization of the alveolar bone, acellular cementum and cellular cementum involved in attachment of the periodontal ligaments. (**B)** Diagram of alveolar bone with osteocytes and lining of osteoblasts on the one surface and periodontal ligaments intersecting the bone surface and the cellular and acellular cementum, with key genes and proteins involved in PDL, such as Periostin, Tenomodulin and Scleraxis transcription factor, two protein involved also in tendon formation in the rest of the body, Nfic transcription factor and Osterix transcription factor. Key matrix proteins, Bsp, Dmp1, and Col12 are components of the attachment apparatus, while Ennp1, TNAP, and Phospho1 are involved in the mineralization of both bone and cementums. A key protein, Sclerostin, produced by osteocytes and cementocytes plays a role in inhibiting the Wnt pathway and the Wnt pathway stimulated many proteins for positive bone and cementum formation through activation of b-catenin that binds Tcf transcription factors and turns select gene on, such as Bmp2 and Bmp4 which stimulate bone formation, and Opg which will inhibit osteoclastogenesis and bone resorption. We now know that Bmp2 induced VegfA from osteoblast is linked to the formation of new microvessels (CD31 and Enmc positive for endothelial cells and Type H microvessels) and associated pericyte like PSC, which are aSMA+ and Osterix+. This coordinate link between capillary microvessel formation and differentiating osteoblast and differentiating PSC is thought to be critical for maintaining the PSC niche and population for future repair as needed. Pth from the circulation or give therapeutically as terapeptide, can play a critical role in inhibiting Sclerostin and stimulating remodeling, We know that the osteocyte under normal physiological conditions, is a major source of the macrophage to osteoclast differentiation RankL factor [10] However, under pathological conditions of inflammatory induced bone loss in periodontal disease, a major local production of RankL comes from activated T and B cells that stimulate macrophage to osteoclast differentiation, leading to bone loss. (**C)** Shows the overall pathways and interactions that are important for healthy periodontium formation and maintenance. For example, we know that Bmp2 can stimulate Osterix through activation of the key transcription factors for bone, Dlx5 and Runx2, while Osterix in turn is linked to directly activating the VegfA gene that then stimulates new microvessels and associated PSCs, ready for a new cycle of regeneration.

We will now discuss in detail evidence for PSC existence and development and detail the potential use of the Sclerostin antibody and parathyroid hormone (Pth) in treatment of bone loss from periodontal disease or other craniofacial trauma.

## Stem Cells of the Periodontium

What are the stem cells for the periodontium, including the alveolar bone, a cellular and cellular cementum, and the tendon-like periodontal ligaments? A variety of studies over the years have identified cells from the dental follicle and the periodontium region of shed teeth that exhibit many of the properties of stem cells with high CD146 and STRO-1 expression [4,5,11]. The dental follicle (DF) progenitors are fairly heterogeneous in their capacity to differentiate towards components of the periodontium and may involve components of HERS [12,13]. Using rigorous in vivo lineage tracing technique, the periodontal stem cells for alveolar bone, cellular cementum, and periodontal ligaments have been identified as a αSMA+ stem/progenitor cell population within the cervical and apical regions of the periodontium [14]. αSMA+ progenitor/stem cells in the bone marrow are, also by lineage tracing, capable of differentiating into osteoblasts [15]. The αSMA+ cells are associated with many smooth muscle cells of the bone and involved in for example vascular contraction-dilation. However, a major pool of these α SMA+ cells in bone and periodontium are associated with the tiny capillaries in bone and periodontium and often referred to as pericytes or mural cells. Pericytes have an intimate relationship with the microvascularity in that loss of pericytes, results in loss of the capillaries of the micro vascular areas in bone and periodontium [16]. With this new system of a aSMA+ promoter driving the Cre recombines with an estrogen receptor mutant that is only activated by Tamoxifen, we can now turn on the Cre postnatal, after root formation is fairly underway, and study lineage tracing in the presence and absence of the Bmp2 gene. We follow the lineage using a Rosa26-loxP-stop-loxP-tdTomato, in which after a Cre event, the stop cassette is removed and the tdTomato gene turns on, and the cell stays red the rest of its life and in the progeny derived from that cell, in this case aSMA+ cells [15].

The Bmp2 gene is thus critical for controlling these aSMA+ stem cells and associated micro vascularization in both the bone marrow and in the periodontium [17,18]. Another excellent candidate marker for stem cells of the periodontium (excluding the gingival and epithelial components) is Osterix+ cells. Osterix is a transcription factor required for long bone formation and for cementum and alveolar bone formation [19]. By lineage tracing studies, the Osterix+ cells can and do overlap with the αSMA+ cells as well as progress to mature osteoblasts [20]. Recently, data also shows Osterix+ cells surrounding the small capillaries in the bone marrow are contributing to the link between angiogenesis to osteogenesis [21]. We have shown that the stem cells (MSC) associated with small capillaries in the bone marrow is greatly reduced in the conditional KO of Bmp2 in osteoblasts [17], and these vascular associated pericytes are a major source of the Bmp2 protein [22]. Recent work has also demonstrated that Osterix+ cells, by lineage tracing in early perinatal life in bone marrow, progress to a stable set of long-lived stromal cells that have characteristics of mesenchymal stem cells [23].

Previously, we have also observed that deletion of Bmp4 in the periodontium leads to major defects in PDL formation and attachment [24]. These observed phenotypes in the cKO of Bmp4 in the periodontium could be related to failure of PDL differentiation and tendon attachment to the bone and cementum surface, since Bmp4 is required for proper attachment of tendon to bone [25].

Recently, the Wnt pathway has also been shown to play a fundamental role in PDL formation and linked to alveolar bone and cementum formation [26]. Lim et al, 2014 show disrupted PDL formation, thinner a cellular cementum, and greatly reduced Osterix+ cells in the periodontium and widen periodontal space. A major negative regulator of Wnt signaling, the Sost gene or Sclerostin protein is produced primarily by osteocytes and cementocytes [27]. Recently, results in the Sost KO mouse, demonstrates that there is a major expansion of the PDL ligaments, cellular cementum and alveolar and basal bone surrounding the teeth [28]. Our recent results have shown that Sost KO in the presence of the Periostin KO rescues many of the alveolar and PDL defects seen in the Periostin single KO [29]. Recently, preclinical studies using the Sclerostin antibody (Scl-Ab), product of the Sost gene, have shown that the Scl-Ab stimulates bone regeneration after experimental periodontitis [30].

## Role of Sclerostin in Periodontal Biology and Use of Antibody to Sclerostin to Treat Periodontal Disease

Sclerostin is glycoprotein that is secreted by cementocytes and osteocytes. The expression of Sclerostin is regulated by the SOST gene located on Chromosome 17 at q12–p21. Sclerostin has similarity to BMP antagonists and binds to the LRP5/6 receptors. It is an antagonist of the canonical Wnt signaling pathway and influences bone and cementum homeostasis [31]. Expression of Sclerostin is also influenced by mechanical loading. Increased mechanical loading *in vivo* has been shown to down regulate the SOST gene resulting in more bone deposition [32]. In the presence of decreased load, the SOST gene is up regulated causing bone resorption [32]

Sost knockout mice show increase alveolar bone and increased cementum [33]. Targeting the SOST gene has been identified as a treatment for bone disorders including osteoporosis, and bone loss as the result of periodontitis. An anti-Sclerostin monoclonal antibody (Scl-Ab) has been developed and has been used in various research studies [34]. Administration of Scl-Ab has been shown to improve bone fracture healing [35] in the rat and nonhuman primate model [34], and restore bone strength and mass in the osteoporotic rat model [36].

Two published studies have demonstrated alveolar bone regeneration in experimental periodontitis [37,30] in ligature induced periodontitis; systemically administered Scl-Ab treated rats had statistically similar bone volume fraction (BVF) and tissue mineral density (TMD) when compared to the periodontally healthy control group. Both BVF and TMD in the Scl-Ab group and healthy control group were significantly higher than the group that received only the antibody vehicle. Local administration of Scl-Ab demonstrated minimum effect on regeneration by analysis with micro CT. Bone formation markers including PINP and osteocalcin in the Scl-Ab group were similar to the periodontally healthy group at 6 weeks. Authors concluded that the systemic administration of Scl-Ab restored alveolar bone mass in the ligature induced periodontitis model [37].

Regeneration of cementum, alveolar bone, and functionally oriented periodontal ligament is the end treatment goal in patients with periodontal disease. After the disease process has been controlled, clinicians can focus on regenerating what has been lost. Current therapies are unable to provide predictable complete regeneration of the periodontium. As demonstrated with the research in the use of Scl-Ab to treat periodontal defects, future therapies could potentially focus on modulating SOST gene expression in patients. In the treatment of periodontal defects, systemic administration of the Scl-Ab has the potential to improve radiographic, histologic, and biochemical indicators of alveolar bone regeneration [37,29,30]. Further research including human trials is needed before the Scl-Ab can be applied to clinical practice.

## Role and Potential Use of Parathyroid Hormone In Periodontal Disease

Parathyroid hormone, secreted by the chief cells in the parathyroid glands, is important regulator of calcium homeostasis and is known to have both anabolic and catabolic effects on bone [38]. The target for parathyroid hormone, the PTH1 receptor is found on osteoblasts and osteocytes. Release of parathyroid hormone indirectly results in increased osteoclastogenesis via its binding to osteoblasts. This in turn leads to an increased expression of RANKL and decreased secretion of OPG by these cells. The corresponding higher RANKL-to-OPG ratio results in an increased differentiation of osteoclasts and therefore greater bone resorption. This catabolic effect of parathyroid hormone is seen at chronic, high levels of hormone. There is, however, an anabolic effect of parathyroid hormone seen at low, intermittent doses. At these levels, parathyroid hormone results in enhanced recruitment, proliferation, and differentiation as well as decreased apoptosis of osteoblasts [39,40].

Parathyroid hormone exerts its bone forming effects through interactions with several different cellular pathways. First, along with Wnt, it increases the commitment of mesenchymal stem cell precursors to the osteoblast cell line [41]. Second, parathyroid hormone binds to PTH1 receptor, a G protein-coupled receptor, and activates phospholipase C, cAMP-dependent protein kinase A, and protein kinase C. The overall effect is an increase in bone formation [42]. Third, the PTH1 receptor is able to activate the Wnt pathway in the absence of Wnt by forming a complex with LRP5/6 after parathyroid hormone binding [43]. This allows for the phosphorylation and stabilization of B-catenin, permitting the Wnt signaling pathway to proceed [44] additionally, the PTH1 receptor is highly expressed on osteocytes. Recent research has shown that parathyroid hormone exerts further effects on the Wnt pathway via these osteocytes receptors by inhibiting both SOST and Dkk1 [45]. By inhibiting SOST and Dkk1, both inhibitors of Wnt, parathyroid hormone effectively allows the continuation of the Wnt pathway, signaling for the proliferation and differentiation of osteoblasts. This interaction has been confirmed in research showing that bone formation induced by parathyroid hormone is diminished in mice that over express SOST [46]. Additionally, mice that is deficient in PTH1 receptor on osteocytes show osteopenic tendencies, with increased SOST expression and decreased Wnt signaling [47].

Research on a possible therapeutic use for parathyroid hormone has focused on teriparatide, a biosynthetic human parathyroid hormone that consists of the first thirty-four amino acids of parathyroid hormone. Teriparatide has already received FDA approval for the treatment of osteoporosis and serves as an alternative to bisphosphates in the prevention of bone density loss. It is currently the only approved anabolic agent available. Significant decreases in non-vertebral and non-vertebral fragility fractures in women treated with teriparatide were shown [48], as well as increases in bone mineral density of osteoporotic patients after treatment with intermittent doses [49]. Papapoulos also showed an increase in serum markers for bone formation, procollagen type 1 aminoterminalpropeptide (P1NP) and carboxy-terminal collagen cross linking (CTX), relative to markers for bone resorption after treatment with teriparatide [50].

Recently, this research has been applied to the oral cavity in the hope of elucidating similar bone formation in patients with periodontal disease. Older studies have already shown that structural integrity of trabecular bone is increased with teriparatide administration [51]. Several animal studies show promise with respect to teriparatide for the treatment of periodontitis. A study that created periodontal defects around dental implants in dogs treated the sites with guided bone regeneration that included teriparatide bound to a synthetic matrix showed potential promise for teriparatide in this application [52]. Another study looking at fenestration defects created in the buccal plate in rats showed enhanced new bone formation, reduced remaining defect size, and increased newly formed cementum-like tissue after intermittent treatment with teriparatide [53]. Potentially most promising, a human clinical trial was completed evaluating the effect of teriparatide on patients with severe chronic periodontitis. Following periodontal surgery, patients received teriparatide injections daily for six weeks. Significant improvements were seen in almost all measured periodontal parameters including gain in linear bone, gain in attachment, and reduction in probing in the test group compared to control [54]. While this is encouraging, further clinical trials are needed to truly determine the long-term effectiveness of this treatment.

## Future Directions, Transcriptomics, and Epigenomics in Periodontal Biology

By understanding the gene and transcription network of how periodontal stem cells differentiates into the 3 major components of the periodontium has great promise to develop specific methods and therapeutics for regeneration of the complete periodontium, at least the mesodermal derived components. With that underlying thought, we have developed a complete transcriptome at 4 different stages of differentiation of a SMA+ PSC, with and without the endogenous Bmp2 gene, using Next Generation Sequencing tools and RNA-seq methods. We have complete transcriptome data on a cementoblast to cementocytes cell model, CM6 [5]. This will allow us to develop a cementocytes specific-enriched gene expression signature.

Just removing the endogenous Bmp2 gene in this *in vitro* system, allowed us to determine a set of ontologies that define the lack of the endogenous Bmp2 gene in these PSC. For example, of the 576 genes reduced in expression, over 51 histone genes and other growth related genes was reduced 2–50 fold. Also, the key differentiation factor for bone and cementum, Osterix is reduced over 5 fold. We generally think of Bmp2 as a differentiation factor, this all depends on the Bmp2-Activin Receptor patterns on the cell at a given stage or time. In this case, we have hard evidence that the endogenous Bmp2 gene is in fact required for the multilayering and slow growth in this a SMA+ PSC differentiation system. We also characterize over 2500 genes that change expression; roughly ½ increase and ½ decrease, between the undifferentiated state and the mineralizing state, with many gene ontologies supporting this differentiation gene set. Most of the genes activated during differentiation are seen in the cells with or without the endogenous Bmp2 gene present. The subset of about 20% that do not respond to differentiation without the endogenous Bmp2 gene is of great interest to us, in determining deeper mechanistic insights into the role of the endogenous Bmp2 gene that cannot be rescued by adding lots of recombinant Bmp2 from the outside.

There is much to be learned in the genome, as we now know that most of the genome at some state or time is in fact transcribed. We sometimes refer to this lack of understanding as the “dark matter of the genome”. But these unknowns offer great potential for a better understanding of many biological processes and opens new windows of treatment and therapy for better health. For example, There is a large collection of transcripts called long-noncoding RNAs (lncRNAs) that do not code for a protein, spread out over the genome (much of the analysis is in human and mouse genomes) and a large fraction of these lncRNAs turn out to be associated with what we define as the regulator regions or enhancers of a given gene that determine if the gene is on or off. These enhancers are determined by chip-seq NGS methods, using antibodies to such histone variants as H3K27ac, where the lysine at position 27 is acetylated by various HAT or histone acetyl transferase enzymatic activities in the activation of these gene regulatory regions or what we call enhancers. These interesting short (100–300bp) transcripts, usually bidirectionally transcribed from both strands of DNA, called enhancer RNAs or eRNAs, have in some cases been shown to be critical for enhancer function for a given gene [55].

For future understanding of PSC differentiation, we have started to map some of these eRNAs by overlap of the transcription of the lncRNA and the H3K27ac chip-seq maps. Currently we have over 4000 enhancers activated within one day of differentiation with rBmp2 treatment, and by 7 days, there are over 5900 new enhancers activated that do not overlap with the undifferentiated set or the early recombinant Bmp2 responsive set of enhancers. A large fraction of these enhancers have differentiation-cell selective associated eRNAs.

By studying these new and interesting regulatory components of PSC differentiation and in many other differentiation systems, we will gain new potential targets for regeneration of new bone and other components that “wear out” because of disease and age.

## Conclusion

We have briefly reviewed current thoughts and data on development, physiology, and pathology associated with stem/progenitor cells of the periodontium. With this new knowledge, thoughts of modulating the Wnt pathway, Bmp2 pathway, and P^th^ signaling were discussed as new clinical paradigms for treating periodontal bone loss as well as loss of craniofacial bone associated with surgery or trauma. The new area of transcriptomics and epigenomics in periodontal biology was introduced from our current work with the SMA+ periodontal stem/progenitor cells (PSC). An overall model of the signaling pathways with their associated transcription factors and matrix components of the periodontium is presented as a guide for future modulation and improved bone formation in the craniofacial region.
